# Expression and characterization of UL16 gene from duck enteritis virus

**DOI:** 10.1186/1743-422X-8-413

**Published:** 2011-08-24

**Authors:** Qin He, Qiao Yang, Anchun Cheng, Mingshu Wang, Jun Xiang, Dekang Zhu, Renyong Jia, Qihui Luo, Zhengli Chen, Yi Zhou, Xiaoyue Chen

**Affiliations:** 1 Institute of Preventive Veterinary Medicine, Sichuan Agricultural University, Wenjiang, Chengdu city, Sichuan, 611130, P.R.China; 2 Avian Disease Research Center, College of Veterinary Medicine of Sichuan Agricultural University, 46# Xinkang Road, Ya'an, Sichuan 625014, P.R. China; 3 Key Laboratory of Animal Disease and Human Health of Sichuan Province, Sichuan Agricultural University, Wenjiang, Chengdu city, Sichuan, 611130, P.R.China

## Abstract

**Background:**

Previous studies have indicated that the UL16 protein and its homologs from herpesvirus were conserved and played similar roles in viral DNA packaging, virion assembly, budding, and egress. However, there was no report on the UL16 gene product of duck enteritis virus (DEV). In this study, we analyzed the amino acid sequence of UL16 using bioinformatics tools and expressed in *Escherichia coli *Rosetta (DE3) induced by isopropy1-β-D-thiogalactopyranoside (IPTG). The recombinant protein was produced, purified using a Ni-NTA column and used to generate the polyclonal antibody against UL16. The intracellular distribution of the DEV UL16 product was carried out using indirect immunofluorescence assay.

**Results:**

In our study, UL16 gene of DEV was composed of 1089 nucleotides, which encoded 362 amino acids. Multiple sequence alignment suggested that the UL16 gene was highly conserved in herpesvirus family. The UL16 gene was cloned into a pET prokaryotic expression vector and transformed into *Escherichia coli *Rossetta (DE3) induced by IPTG. A 60kDa fusion protein band corresponding to the predicted size was produced on the SDS-PAGE, purified using a Ni-NTA column. Anti-UL16 polyclonal sera was prepared by immunizing rabbits, and reacted with a band in the IPTG induced cell lysates with an apparent molecular mass of 60 kDa. In vivo expression of the UL16 protein in DEV infected duck embryo fibroblast cells (DEFs) was localized mostly around perinuclear cytoplasmic area and in cytosol using indirect immunofluorescence assay.

**Conclusions:**

The UL16 gene of DEV was successfully cloned, expressed and detected in DEV infected DEFs for the first time. The UL16 protein localized mostly around perinuclear cytoplasmic area and in cytosol in DEV infected DEFs. DEV UL16 shared high similarity with UL16 family members, indicating that DEV UL16 many has similar function with its homologs. All these results may provide some insight for further research about full characterizations and functions of the DEV UL16.

## Background

Duck viral enteritis (DVE), an acute and contagious disease, is highly lethal in all ages of birds from the order Anseriformes (ducks, geese, and swans). This disease is characterized by vascular lesions and tissue hemorrhage, as well as gastrointestinal, lymphatic, and nervous impairments [1-3]. Duck enteritis virus (DEV) is the causative agent for DVE and was first recorded in Holland in 1923 [4], more outbreaks were reported in the North America [5], Canada [6], France [7] and China [8] et al.

According to the Eighth International Committee on Taxonomy of Viruses (ICTV), DEV (anatid herpesvirus I) is a member of subfamily Alphaherpesvirinae of the family Herpesviridae but not assigned to any genus [9]. Like other alphaherpesviruses, DEV is a large, enveloped virus with four structural components including linear double strand DNA, an icosahedral capsid, an amorphous tegument and a bilayer lipid envelope. In recent years, a lot of DEV genes have been identified and reported, such as glycoprotein B gene, glycoprotein E gene, thymidylate kinase gene, dUTPase pyrophosphatase gene et al [10-12].

The UL16 genes of alphaherpesviruses encode tegument proteins, which are conserved throughout the herpesvirus family. Previous researches have indicated that the UL16 protein of herpes simplex virus type 1 (HSV-1) is not required for viral replication in cell culture, and its function may be in viral DNA packaging, virion assembly, budding, and egress [13-16]. Firstly, UL16 protein binds to nuclear capsids during nuclear egress. Secondly, UL16 protein attaches to DNA- containing C-capsids in the cytoplasm prior to their arrival at the trans-Golgi network (TGN) for maturation budding. Thirdly, UL16 protein interacts with UL11 which is membrane-bound fastened capsids to the menbrane and drived the budding process [17]. During budding events, the UL16 protein provided abridging function between the capsid and the membrane [18-21]. Interaction of the UL16 tegument protein with the capsid of herpes simplex virus is dynamic, with a binding and release mechanism that is regulated by pH and likely involved scysteines. There are 20 cysteines in UL16 protein, including five cysteines that are conserved within a putative zinc finger [22]. After the capsid budding into the TGN, capsid and tegument proteins also encounter an oxidizing and a low pH environment, which is conducive to trigger conformational changes and disulfide bond formation [23,24]. Subsequently, the virions release to the extracellular medium where the pH returned to 7.4. In the extracellular medium, the interaction of UL16 with capsid is unstable and UL16 protein releases from the capsid to promote capsid to reenter the next cell [16].

However, the structure and function of DEV UL16 tegument protein remain unknown. In this study, we report the identification, cloning and molecular characterization analysis of the DEV UL16 gene and its prokaryotic expression. These works may provide some insights for further research about characterizations and functions of DEV UL16.

## Results

### Identification and Molecular characteristics of DEV UL16 gene

**Figure 1:**
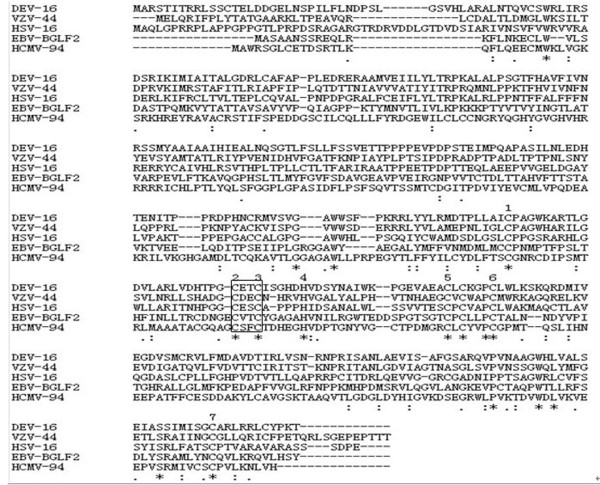
**Amino acid sequence comparison between the putative proteins encoded by DEV UL16 and it homologs: HSV-1 UL16, VZV ORF44, EBV BGLF2, and HCMV UL94**. Sequences were aligned with the Clustalx1.8 software. Absence of amino acid is shown by dash '-' in the sequences while '*', ':', and '.' indicate identical amino acid residues, conserved residues and semi-conserved residues in all sequence used in the alignment respectively.

The open reading frame (ORF) of DEV UL16 gene (EU195095) consisted of 1089 bp and potentially encoded a protein of 39.87 kDa, consisting of 362 amino acids and with an isoelectric point of 7.73. Computer analysis showed that the UL16 amino acid sequence contains 16 possible sites for phosphorylation. Five casein kinase II, one cAMP- and cGMP- dependent protein kinase, five protein kinase C phosphorylation sites and four potential N-linked myristoylation sites were present along the amino acid sequence. Transmembrane and signal peptide regions were not found. The analytical result with the program PredictNLS showed this protein did not contain a nuclear localization sign (NLS). The prediction result with the online program LOCtree sugguested that the DEV UL16 protein was predicted to mostly localizated in cytoplasm. These results were consistent with the previous predictions [25]. At the same time, a conserved domain of herpes_UL16 superfamily was detected by the conserved domain database (CDD) in the deduced 362-aa protein. This provided further evidence that the deduced protein is UL16 protein. Previous studies indicated that the UL16 protein was conserved throughout the herpesvirus family. Figure 1, showing the UL16 family members of herpesviruses revealed that they contain at least six conserved cystein residues as well as one conserved histidine residue in the protein sequences, and DEV UL16 shares identities of 38.1% with HSV-1, 37.2% with varicella zoster virus (VZV) ORF44, 19.8% with epstein-barr virus (EBV) BGLF2 and 17.8% with human cytomegalovirus (HCMV) UL94, suggesting a potential related function.

### Cloning and Construction of the pET32b -UL16

**Figure 2:**
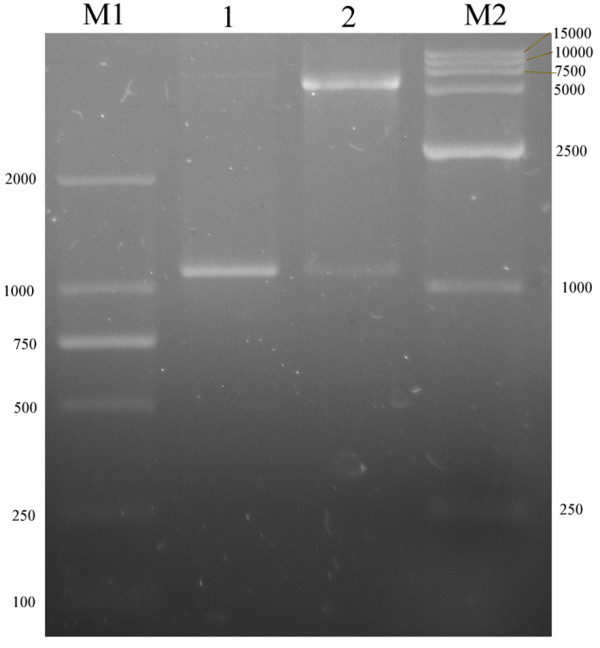
**Identification of recombination vector pET 32b (+)-UL16 by restriction enzymes digestion**. Lane M1, DNA marker; Lane 1, PCR products from pET 32b(+)-UL16; Lane 2, recombination plasmid pET 32b(+)-UL16 was digested with two restriction enzymes HindIII and XhoI; Lane M2, DNA marker.

A predicted 1098bp product containing the entire ORF of DEV UL16 was amplified using PCR from DEV CHv strain. The sequencing result of the resulting clone pMD18-T-UL16 was consistent with the sequence of the ORF found in the genomic library of DEV. The constructed pMD18-T-UL16 was cut with HindIII and XhoI, and the insert was ligated into pET32b (+) vector precut with the same enzymes. The resulting pET32b-UL16 was verified by restriction enzymes analysis (Figure 2).

### Expression and purification of the Recombinant Protein

**Figure 3:**
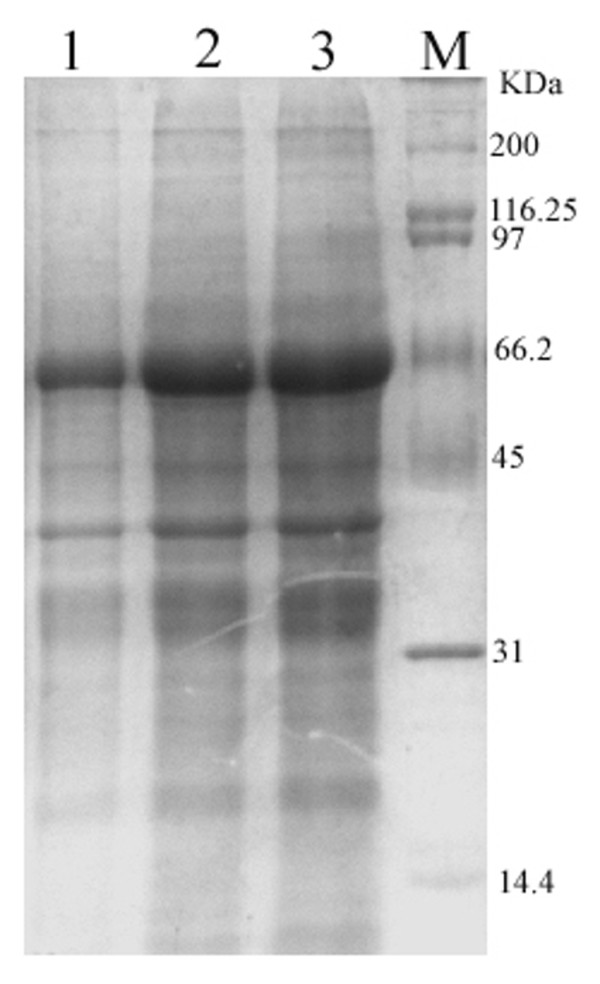
**SDS-PAGE analysis of the expression results of recombinant plasmid pET32b-UL16 in different temperature from Rossetta(DE3)**. Lane M = Protein marker; lanes 1-4 = cells were grown respectively at 25, 30, 37°C after induction with IPTG.

**Figure 4:**
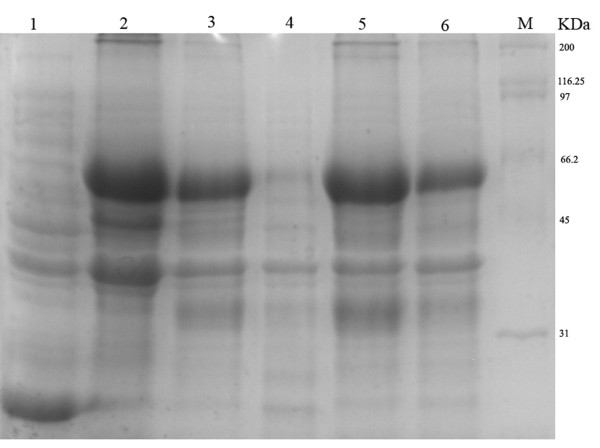
**SDS-PAGE analysis of different time course of recombinant plasmid pET32b-UL16 production from Rossetta(DE3)**. Lane M = Protein marker; lanes 1 = the plasmid pET32b (+) was induced with IPTG; Lane 4 = the recombinant plasmid pET32b-UL16 was uninduced; Lane 2, 3, 5, 6 = cells were grown respectively for 6, 8, 4 and 2 h after induction with IPTG.

**Figure 5:**
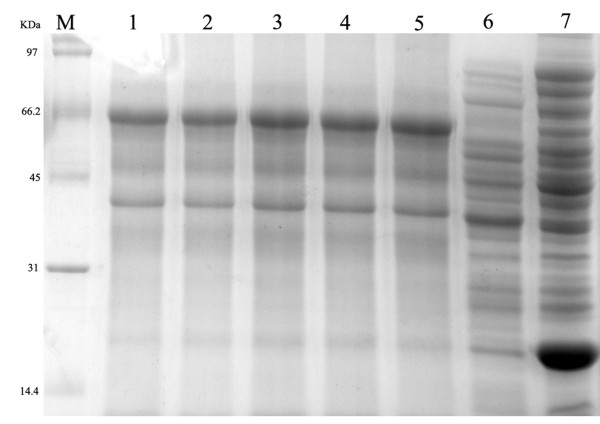
**Production of recombinant plasmid pET32b-UL16 from Rossetta(DE3) in different IPTG concentrations**. Lanes 1-6 = cells were grown and induced at 1.0, 0.8, 0.6, 0.4, 0.2 and 0.0 mmol/l IPTG, Lane 7 = the plasmid pET32b (+) was induced with IPTG.

**Figure 6:**
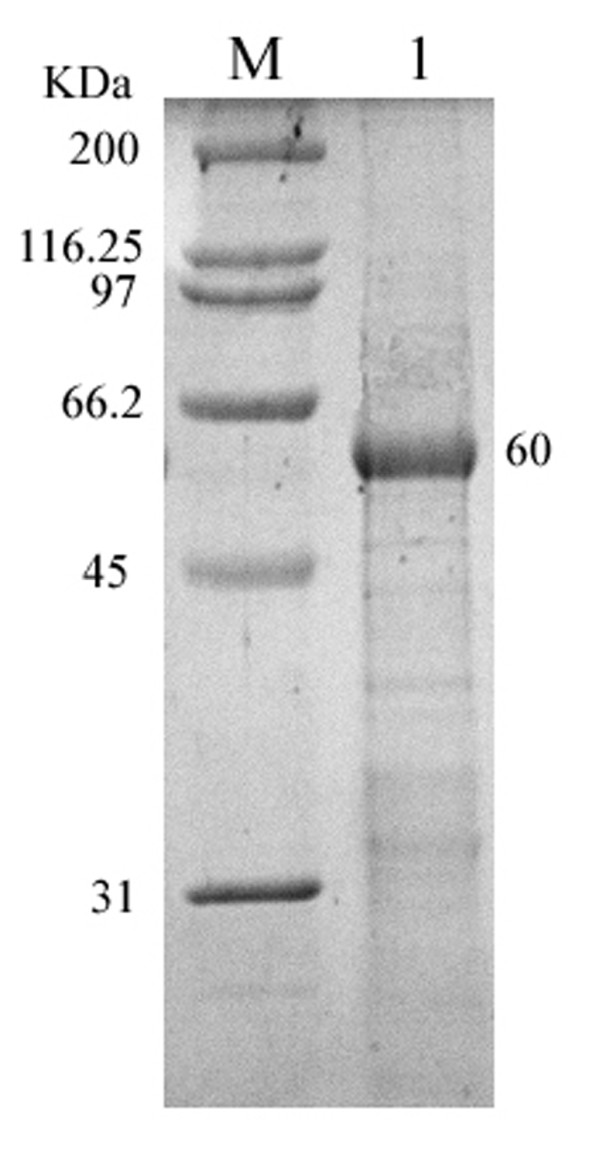
**SDS-PAGE analysis of the purity of recombinant plasmid pET32b-UL16 from Rossetta (DE3)**. Lane M = Protein marker; lanes 1 = recombinant protein from Rossetta (DE3) purified by the Ni^+^-NTA agarose gel.

The recombinant expression plasmid pET32b-UL16 was transformed into *Escherichia coli *Rossetta (DE3). To obtain a highly expressed level of UL16 protein, we tried optimizing expression conditions by using different temperatures (25, 30, 37°C), different IPTG concentrations (0.0, 0.2, 0.4, 0.6, 0.8, 1.0 mM), and different incubation times (0, 2, 4, 6, 8 h). We found that the expression level of synthesized pET32b-UL16 at 37°C was slightly more than at 25°C and 30°C (Figure 3). While incubation time was increased, the expressed protein was increased, too (Figure 4). The different concentrations of IPTG showed no apparent increase in the expressed protein (Figure 5). A 60 KDa fusion protein was highly expressed after induction at 37°C for 6 h with 0.2 mM IPTG and purified using the Ni-NTA column by imidazole (Figure 6).

### Preparation and specificity of anti-UL16 protein antiserum

**Figure 7:**
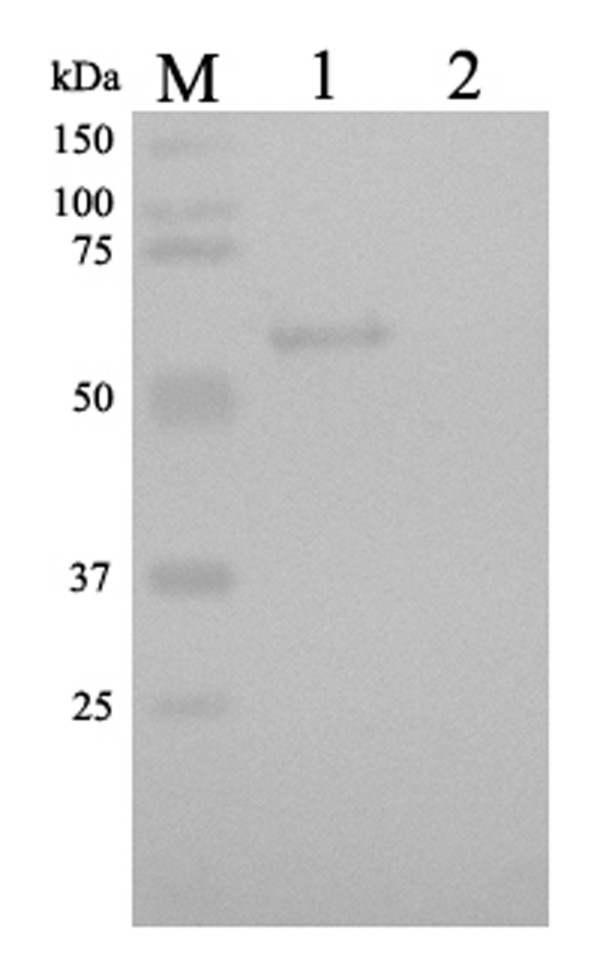
**Western blotting identification of recombinant protein UL16 expressed in *Escherichia coli *Rossetta (DE3) with the rabbit serum of anti-DEV UL16 IgG**. lane 1 = expression of recombinant plasmid pET32b-UL16 induced by IPTG; Lane 2 = Expression of recombinant plasmid pET32b-UL16 uninduced *Escherichia coli *Rossetta (DE3) cell lysate.

The anti-UL16 protein antiserum was prepared as described in Methods. Western blotting experiment was performed to examine the reactivity and specificity of the UL16 antiserum. Figure 7 showed that the UL16 antiserum reacted with a band in the IPTG induced cell lysates with an apparent molecular mass of 60 kDa (lane 1). In contrast, no specific band was shown in uninduced cell lysates (lane 2). This result demonstrated that the antisera against DEV UL16 protein was specific.

### Subcellular location of the UL16 product in DEV-infected cells

**Figure 8:**
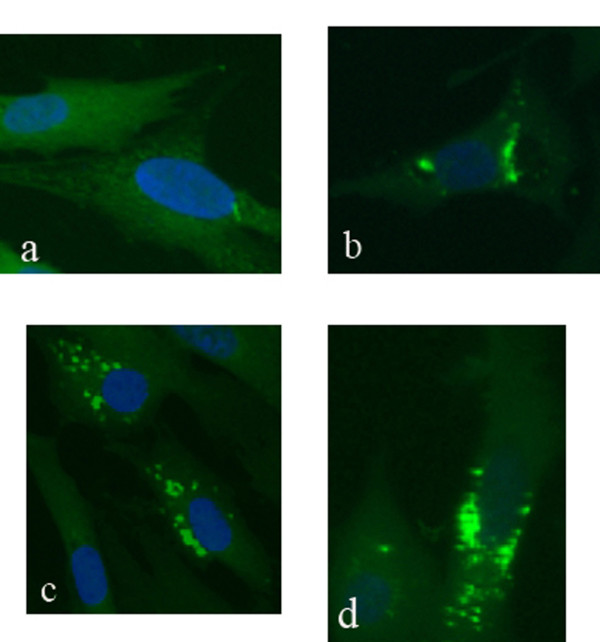
**Intracellular localization of the UL16 protein in DEV CHv infected DEFs**. Mock- and DEV CHv infected cells were fixed at the indicated times postinfection and processed for indirect immunofluorescence. Mock-infected cells were fixed after 36 h incubation (a); DEV CHv infected cells were fixed at 18(b), 24(c), and 36 h postinfection (d). ×400.

The intracellular distribution of UL16 protein was examined by indirect immunofluorescence assays as described in Materials and Methods. As shown in Figure 8b, the UL16 protein appeared in the perinuclear cytoplasm area at 18 h postinfection, and in the cytoplasm at 24, 36 h postinfection (Figure 8c, d). These fluorescences were absent in mock-infected cells (Figure 8a) and no significant fluorescence was observed with the preimmune serum (not shown).

## Discussion

In this study, we reported the cloning, expression and characterization of the UL16 gene from DEV CHv strain. Sequence analysis indicated that the protein, encoding by UL16 gene, has no transmembrane helix structure and signal peptide sequence the same as described previously [25], which provided some information for its expression. In addition, multiple sequence alignment of the UL16 proteins revealed that DEV UL16 shared high similarity with UL16 family members and contained at least six conserved cystein residues as well as one conserved histidine residue in the protein sequences. Moreover, previous studies indicated that the UL16 homologs contain seven conserved cystein residues as well as two conserved histidine residues in the middle of the ORFs, suggesting the presence of a possible zinc and/or nucleic acid binding function for these homologs [22]. All of these suggest that the UL16 protein may serve the same role as homologous in HSV-1.

Subsequently, DEV UL16 gene had been expressed successfully in *Escherichia coli *strain Rossetta (DE3), and polyclonal antibody raised against the recombinant UL16 from rabbit was prepared. Using this antibody, we found that the UL16 fusion protein was approximate 60kDa and localized in perinuclear cytoplasmic region and cytoplasm of infected cells. The homologous HSV-1 and HSV-2 proteins have been found to be primarily nuclear at early times postinfection. The localization of UL16 changes to a mostly punctate perinuclear region and cytoplasm at later times postinfection [14,15]. Barbara G. Klupp and his collaborators [20] argued that, in Pseudorabies Virus (PRV), there was a putative nuclear localization signal between aa 261 and 267 of UL16, but UL16 specific fluorescence was detected mainly in the cytoplasm of infected cells. There might be a difference among HSV-1, HSV-2 and DEV. In our study, the UL16 protein was not found in the nuclear. On the other hand, by the analysis of bioinformatics, no NLS of the DEV UL16 protein was identified and its localization prediction showed the protein was located mostly in cytoplasm. This prediction result was supported by our subcellular localization result. The HSV-1 UL16 protein was known as a tegument protein and its function may be involved in viral DNA packaging, virion assembly, budding, and egress by providing an interaction with the membrane-bound UL11 protein and the UL21 protein [13,15,16]. The UL21 protein was associated with capsids and microtubules protein [17,20]. In cell nuclei, nucleocapsids undergo movement toward the nuclearenvelope, possibly along actin filaments through interacting with motormyosin V [26,27]. After the nucleocapsids budding into the TGN, where the nucleocapsid is maturating, the UL16 protein bounds to the leucine-isoleucine (LI) motif and the acidic cluster (AC) of UL11 protein. In the TGN, capsid and tegument proteins also encounter an oxidizing and mildly acidic medium (pH5.0 to 5.5) environment, which is conducive to disulfide bond formation. The UL16 releases from capsids during egress through low-pH compartments of the cell [16,21,23,28,29]. In the absence of murine gammaherpesvirus 68 (MHV-68) ORF33 (UL16 homologue), immature virions were restrained in a state interacting with actin and glycoproteins gB, failed to release the infectious virions [30]. Cysteines of the UL16 protein sequence were required to perform these functions. UL16 homologs have revealed that they contain seven conserved cysteine residues as well as two conserved histidine residues in the middle of the ORFs [22]. Moreover, Pei-Chun Yeh et al [21] presumed that the HSV-1 UL16 might be an enzyme that utilizes one or more free cysteines in its active site to catalyze the formation and breakage of disulfide bonds. The protein disulfide isomerases, which catalyze the formation and breakage of disulfide bonds, generally have a C-X-X-C motif in their active site. The DEV UL16 amino acid contains 13 cysteine residues, including six that are conserved throughout the herpesvirus family. We also found a C-X-X-C motif among the conserved residues of DEV UL16 (Figure 1). DEV UL16 may have the same funtions in virion assembly, budding, and egress by interacting with other proteins. Hence, the DEV UL16 is needed to further study.

## Conclusion

In conclusion, we report firstly the cloning, expression and the initial characterization of DEV UL16 gene in this study. The UL16 proteins localized mostly around perinuclear cytoplasmic area and in cytosol in DEV infected DEFs. DEV UL16 shared high similarity with UL16 family members, indicating that DEV UL16 many has similar function with its homology. All these results may provide some insight for further research about full characterizations and functions of the DEV UL16.

## Materials and methods

### Viruses, cells and viral DNA extraction

DEV CHv strain was provided by the Key Laboratory of Animal Disease and Human Health of Sichuan Province, and propagated in DEFs. Growth medium consisted of MEM medium (Gibco-BRL) supplemented with 10% calf serum. The maintenance medium consisted of MEM medium (Gibco-BRL) supplemented with 2% calf serum. The infected DEFs were harvested when the cytopathic effect (CPE) was above 80%. After three freeze-thaw treatments, the mixture was subjected to centrifugation at 10,000 g for 30 min. The supernatant was incubated with genomic DNA extraction buffer (10 mM Tris-HCl [pH 8.0], 1 mM EDTA, 1% sodium dodecyl sulfate and 500 ug/mL proteinase K) at 56°C for 3 h. After extracting twice with phenol-chloroform and precipitating with ethanol, the viral DNA was dissolved in TE buffer (10 mM Tris-HCl [pH 8.0], 1 mM EDTA).

### PCR, T-Cloning and sequencing

The primers were designed according to the UL16 gene (GeneBank Accession no. EU195095) and used to amplify a fragment (1098 bp) containing the complete ORF of DEV UL16 gene as previously study [25]. Forward primer (P1) 5'-AAGCTTATGGCTCGCAGTACTATTA-3' and the reverse primer (P2) 5'-CTCGAGGACAGTATATTATGTTTTGG-3' containing the HindIII and XhoI restriction sites (underlined), respectively. The two primers were synthesized by TaKaRa (Dalian, China) and dissolved in ultrapure water to 10 pmol ul^-1 ^concentration for use. A 50 μl PCR reaction contained the following: 25 μl 2 × Taq PCR MasterMix (Takara Ltd. Co.), 1.5 μl of each primer, 0.5 μg DNA template and 11 μl ultrapure water. PCR was carried out using the Biometra PCR equipment (Germany) and initiated with an incubation step at 95°C for 5 min, followed by 25 cycles of denaturation at 95°C for 30 s, annealing for 30 s at 53.6°C, and extension at 72°C for 30 s, then with a final extension step at 72°C for 10 min. The amplified DNA products were electrophoresed on a 1.0% (w/v) agarose gel, and analyzed using gel imaging system (Bio-Rad, USA). The amplified products were sent to Takara Ltd. Co. for construction of T-clone pMD18-T-UL16 and sequencing of the UL16 insert.

### Characterization on the bioinformatics of the UL16 protein of DEV

The result of sequencing analyzed using the ORF Finder software program to uncover possible ORFs. The predicted ORFs were subsequently subject to searching conserved domain in the CDD [31] to verify gene identification. The nucleotide sequences characterization and its encoding protein of the DEV UL16 gene were analysed by DNASTAR software and multiple sequence alignment was performed with CLUSTAL-X software. The signal peptides analysis, the possible nuclear localization signal and the subcellular localization of the UL16 protein were online performed with SignalP version 3.0 (http://www.cbs.dtu.dk/services/SignalP/), PredictNLS program (http://www.rostlab.org/services/predictNLS/), LOCtree program (http://www.rostlab.org/cgi/var/nair/loctree/query), respectively [32-34]. The functional sites were predicted by PROSCAN against PROSITE database (http://npsa-pbil.ibcp.fr/).

### Expression of the His-tagged UL16 fusion protein

We expressed the fusion protein as described previously [35]. The T-clone plasmid, pMD18-T- UL16, was digested with the endonucleases HindIII and XhoI, and the UL16 target sequence was subcloned into the same multicloning sites of pET32b (+) (Invitrogen). The recombinant plasmid pET32b-UL16 was transformed into *Escherichia coli *Rossetta (DE3). The transformed bacterias were grown on LB plates with 100 ug/ml ampicillin at 37°C for 24 h. A single colony from the culture was grown in LB medium with ampicillin to an optical density (OD_600_) of 0.6. The fusion protein was induced by the addition of 0.2 mM isopropyl-β-D-thiogalactopyranoside (IPTG) treatment for 6 h. The cells were harvested by centrifugation at 8,000 rpm and 4°C for 10 min and lysed in 5 × SDS-PAGE loading buffer (0.25 M Tris-HCl[pH 6.8], 50% glycerol, 10% SDS, and 0.05% bromophenol blue, with 0.05% β-mercaptoethanol). Then, the cell lysates were boiled for 10 min, centrifuged at 12,000 rpm for 10 min. Total cell proteins were analyzed by SDS-PAGE using 12% polyacrylamide gel. Uninduced recombinant clone and *Escherichia coli *Rossetta (DE3) host cells (with and without IPTG) were used as controls. Briefly, the gel was stained with Coomassie brilliant blue R-250 1 h and destained in 6% acetic acid until a clear background was seen.

To increase the production of the recombinant protein, culture conditions for expression were optimized in terms of different temperatures (25, 30 and 37°C), concentrations of IPTG (0, 0.2, 0.4, 0.6, 0.8, 1.0 mM), and durations of induction (0, 2, 4, 6 and 8 h). Protein expression was assessed by SDS-PAGE, as described above.

### Purification of the Recombinant Protein

The induced cells were centrifuged at 8,000 rpm and 4°C for 10 min, resuspended in 20 mM Tris-HCl pH 8.0 containing 1.0 mg/ml lysozyme at -20°C overnight. The bacterias were ultrasonically lysed (UltrasonicProcessor-500), and the lysates were centrifuged at 10,000 rpm (Beckman F2040) 4°C for 10 min. The collected pellets contained the fusion protein in inclusion bodies. The pellets were washed in buffer (2 M urea in PBS) six times for preliminary purification and resuspended in 8 M urea. The preliminarily protein mixture was further purified on a Ni^2+ ^chelating column (BioLogic DuoFlow™ Chromatography System) according to the manufacturer's instructions.

### Preparation of antisera against the UL16 protein

To raise antisera against the UL16 protein according to the protocol of Xiang et al [36], the purified protein was mixed with an equal volume of Freund's complete adjuvant (Sigma) and intradermally injected into rabbits at a dose of 0.5 mg protein per rabbit. Additional twice hypodermic inoculations of 1.0 mg purified recombinant protein per rabbit with Freund's incomplete adjuvant were performed after two weeks and three weeks. Subsequently, each rabbit was intravenously immunized with 0.1 mg of the purified recombinant protein. Two weeks after the last inoculation, the rabbits were exsanguinated to collect the antisera. IgG was purified from the antisera with DEAE-Sepharose column (Bio-Rad) and stored at -70°C until further use.

### Western-blot Assay

To characterize the antigenicity of the recombinant fusion protein, Western blot analysis was performed according to a standard procedure [37] using the purified rabbit anti-UL16 IgG. The recombinant fusion protein was resolved on 12% (w/v) SDS-PAGE and electro-blotted onto polyvinylidene difluoride PVDF membrane using wet transfer method. The membrane was then blocked in 3% BSA in PBS-T (0.2% Tween-20 in PBS, PH 7.4) for 1-2 h at 37°C. After washing three times with PBS-T, the membrane was incubated with rabbit anti-UL16 IgG at a dilution of 1:100 with 0.5% BSA in PBS-T overnight at 4°C. The membrane was then washed three times with PBS-T, and further incubated with horseradish peroxidase-labeled goat anti-rabbit IgG (Bio-Rad) at a dilution of 1: 3,000 for 1 h at 37°C. The membrane was then washed three times with PBS-T and reacted with diaminobenzidine substrate buffer. Color development was terminated by thorough washing in distilled water.

### Indirect immunofluorescence assays of infected cells

For indirect immunofluorescence tests, DEFs were grown on coverslips and mock infected or infected with DEV CHv at a multiplicity of infection of 1 PFU/cell. After 18, 24, and 36 h postinfection, the coverslips were washed with PBS three times and harvested by fixation in 4% formaldehyde for 15 min at room temperature. After washing three times with PBS, the cells were permeabilized with 0.2% Triton X-100 (v/v in PBS) for 30 min at 4°C. The coverslips were washed in PBS containing 0.1% Tween-20 and then blocked in 4% BSA in PBS for 1 h at 37°C. Subsequently the coverslips incubated with rabbit anti-UL16 IgG (diluted 1: 100 with 1% BSA in PBS) for overnight at 4°C. Then, the cells were reacted with FITC-conjugated goat anti-rabbit immunoglobulin (diluted 1: 100 in PBS) for 1 h at 37°C. The coverslips were again washed 3 times. The cell nucleus were visualized by DAPI counter-staining (5 ug/ml, Beyotime) and the coverslips mounted onto glass slides with a drop of glycerol. Fluorescent images were viewed and recorded with a Nikon 80i (Nikon, Japan) fluorescence microscope.

## Competing interests

The authors declare that they have no competing interests.

## Authors' contributions

QH and QY carried out most of the experiments and wrote the manuscript. ACC and MSW have critically revised the manuscript and the experimental design. DKZ, JX, RYJ, LQH, CZL, YZ and XYC helped in experiments and drafted the manuscript. All authors read and approved the final manuscript.
